# Nutrients Can Enhance the Abundance and Expression of Alkane Hydroxylase CYP153 Gene in the Rhizosphere of Ryegrass Planted in Hydrocarbon-Polluted Soil

**DOI:** 10.1371/journal.pone.0111208

**Published:** 2014-10-31

**Authors:** Muhammad Arslan, Muhammad Afzal, Imran Amin, Samina Iqbal, Qaiser M. Khan

**Affiliations:** 1 Soil and Environmental Biotechnology Division, National Institute for Biotechnology and Genetic Engineering, Faisalabad, Pakistan; 2 Agricultural Biotechnology Division, National Institute for Biotechnology and Genetic Engineering, Faisalabad, Pakistan; 3 Earth Sciences Department, King Fahd University of Petroleum and Minerals, Dhahran, Saudi Arabia; Oak Ridge National Laboratory, United States of America

## Abstract

Plant-bacteria partnership is a promising strategy for the remediation of soil and water polluted with hydrocarbons. However, the limitation of major nutrients (N, P and K) in soil affects the survival and metabolic activity of plant associated bacteria. The objective of this study was to explore the effects of nutrients on survival and metabolic activity of an alkane degrading rhizo-bacterium. Annual ryegrass (*Lolium multiflorum*) was grown in diesel-contaminated soil and inoculated with an alkane degrading bacterium, *Pantoea* sp. strain BTRH79, in greenhouse experiments. Two levels of nutrients were applied and plant growth, hydrocarbon removal, and gene abundance and expression were determined after 100 days of sowing of ryegrass. Results obtained from these experiments showed that the bacterial inoculation improved plant growth and hydrocarbon degradation and these were further enhanced by nutrients application. Maximum plant biomass production and hydrocarbon mineralization was observed by the combined use of inoculum and higher level of nutrients. The presence of nutrients in soil enhanced the colonization and metabolic activity of the inoculated bacterium in the rhizosphere. The abundance and expression of CYP153 gene in the rhizosphere of ryegrass was found to be directly associated with the level of applied nutrients. Enhanced hydrocarbon degradation was associated with the population of the inoculum bacterium, the abundance and expression of CYP153 gene in the rhizosphere of ryegrass. It is thus concluded that the combination between vegetation, inoculation with pollutant-degrading bacteria and nutrients amendment was an efficient approach to reduce hydrocarbon contamination.

## Introduction

Various human activities, such as exploration, extraction, refining, storage and transportation of petroleum oil and its products, are polluting the environment with hydrocarbons. The hydrocarbons in soil and water adversely affect plant health and microbial population. Furthermore, hydrocarbons exert toxic effects to animals and humans [1]. Therefore, the removal of hydrocarbons from soil and water is one the main issues in field of environmental sciences [2].

Phytoremediation, i.e., use of plants for the remediation of contaminated soil and water, is one of the most suitable technology for the cleanup of contaminated environment [3]. However, the presence of hydrocarbons in soil reduces plant growth and consequently phytoremediation efficiency [4], [5]. To improve plant growth and phytoremediation of soil contaminated with hydrocarbons, the combined use of suitable plants and hydrocarbon-degrading bacteria has been recently proposed [6], [7]. In the root zone, plants and their associated rhizobacteria form synergistic relationships with each other. Plant roots releases nutrients that enhance the proliferation, colonization and activity of microorganisms in the rhizosphere [8], [9]. Cébron et al. (2009) also proposed the similar effects of vegetation on bacterial community structures especially about the polycyclic aromatic hydrocarbon degraders [10]. In return, rhizobacteria reduce phytotoxicity of hydrocarbons by the virtue of their pollutant-degrading pathways and metabolic activities [2]. In addition to this, rhizobacteria also produce plant growth promoting hormones which promote the plant growth and development [11].

Degradation of hydrocarbon depends upon the abundance and expression of hydrocarbon-degrading genes in soil [6], [12], [13]. Therefore, it is important to analyze the levels of abundance and expression of pollutant-degrading genes in the contaminated soil during phytoremediation [14].

During phytoremediation of hydrocarbon contaminated soil, competition for nutrients between plants and their associated microorganisms enhances. The hydrophobic nature of hydrocarbons reduces the availability of nutrients to the plants and microorganisms [15], [16]. Hence, nutrients such as nitrogen, phosphorus and potassium, act as limiting factors for plant growth and microbial proliferation [17]. Furthermore, nutrients level in soil affects mineralization of organic pollutants [18], [19]. However, the effects of nutrients on the abundance and expression of catabolic genes in hydrocarbon contaminated soil have been rarely evaluated. The objective of the current investigation was to evaluate the effects of two different nutrient levels on the abundance and expression of alkane-degrading gene (CYP153) in the rhizospheric soil of ryegrass grown in hydrocarbon-contaminated soil.

## Materials and Methods

### 1. Bacterial strain

*Pantoea* sp. strain BTRH79, previously isolated and characterized for hydrocarbon degradation [20] and ACC deaminase activity [21], was used in this study. This strain possessed alkane hydroxylase CYP153 gene with a substrate range between C_4_–C_16_. *Pantoea* sp. strain BTRH79 was cultured in LB broth for overnight and harvested by centrifugation, further washed and suspended in autoclaved 0.9% (w/v) NaCl solution. This bacterial suspension was used as inoculum.

### 2. Experiment setup

Agricultural loamy soil, after passing through 2 mm sieve and spiking with diesel (15 g kg^−1^ soil), was filled into plastic pots (1.5 kg soil pot^−1^) and subsequently pots were placed in the greenhouse. Spiking was done by thoroughly mixing the appropriate amount of diesel fuel (15 g) with fresh soil designated for one pot. The water holding capacity of the soil was 45.3 g 100 g^−1^. Different treatments were:

1. Ryegrass vegetated in uncontaminated soil without bacterial inoculation (control);
2. Ryegrass vegetated in uncontaminated soil with bacterial inoculation (control);
3. Soil contaminated with diesel without vegetation but with inoculation;
4. Ryegrass vegetated in diesel contaminated soil without bacterial inoculation;
5. Ryegrass vegetated in diesel contaminated soil with bacterial inoculation;
6. Ryegrass vegetated in diesel contaminated and inoculated soil with lower levels of nutrients (N, P and K); and
7. Ryegrass vegetated in diesel contaminated and inoculated soil with higher levels of nutrients.

Each treatment was triplicated. Surface-sterilized ryegrass seeds were planted, i.e. 100 seeds pot^−1^. After one week of seed germination, poor growing seedlings were removed and a population of 80 plants per pot was maintained. Two different levels of nutrient, i.e., low (N_80_P_50_K_30_) and high (N_120_P_80_K_60_) were applied for two times with an interval of 5 days after one week of germination. Nitrogen was provided in the form of urea while phosphorus and potassium were added as KH_2_PO_4_. Finally, the amount of phosphorus was balanced with Na_2_HPO_4_. For higher dose of nutrients, 3.85 g urea, 3.138 g KH_2_PO_4_ and 2.29 g Na_2_HPO_4_ were added in each pot. Whereas for lower dose of nutrients, 2.57 g urea, 1.56 g KH_2_PO_4_ and 1.81 g Na_2_HPO_4_ were added in each pot.

### 3. Plant growth

Plants were cut 1 cm above-ground after 100 days of sowing and shoot length were determined. Rhizosphere soil samples were obtained by gently agitating the roots. Bulk soil samples were collected and stored at −80°C for further analysis. Roots were washed several times with tap water and then with sterile distilled water. Fresh and dry biomass of shoot and root were estimated as explained previously [21]. Furthermore, different agronomic characters such as average shoot length, average diameter, and network length were measured using ImageJ software [22]. The plants were selected using ranked set sampling procedure [23], [24].

### 4. Enumeration of inoculated bacterium

The population of *Pantoea* sp. strain BTRH79 in the rhizosphere soil was enumerated by viable cell count procedure. Soil slurry was prepared by mixing 3 g of rhizospheric soil in 9 mL of 0.9% (w/v) NaCl solution. After settlement of soil particles, supernatant was taken in sterilized falcon tubes and their ten-fold serial dilutions (up to 10^−3^) were plated on M9 medium containing diesel as a sole source of carbon. Finally, the colony forming units (CFU) were enumerated by using Fotodyne's TotalLab Quant Analysis software [25]. From each treatment, ten colonies were randomly picked and the identity of the isolates with the inoculant strain was confirmed by restriction fragment length polymorphism analysis [21], [26].

### 5. Quantification of the abundance and expression of CYP153 gene

For the quantification of gene abundance and gene expression, DNA and RNA were isolated from the rhizospheric soil using FastDNA Spin kit and FastRNA Pro Soil-Direct kit (Biomedical, USA), respectively. For expression analysis, cDNA was transcribed from the RNA using RevertAid First Strand cDNA Synthesis Kit.

The primers, P450fw1 (5′-GTSGGCGGCAACGACACSAC-3′) and P450rv3 (5′-GCASCGGTGGATGCCGAAGCCRAA-3′) were used for the amplification of the CYP153 gene in qPCR [27]. Gene abundance was determined by using real-time PCR, iCycler (IQ5) (BioRad). Reaction mixtures (25 µL) contained 12.5 µL of Q-Mix (Biorad), 2.5 µL of 10 mg/ml BSA, 1.0 µL DMSO, 0.5 µL of each forward and reverse primer, 5.5 µL of PCR water, 2.5 µL of template DNA and RNase-free water. Temperature profile began with 4 minutes at 94°C followed by 40 cycles of 94°C for 30 seconds, 58°C for 30 seconds, and 72°C for 45 seconds followed by a melt curve from 50 to 100°C. Besides melting curve analysis, PCR products were examined on 2% agarose gels. No primer-dimers were detected. Primer-dimer is a potential by-product and in quantitative PCR it may interfere with accurate quantification. Serial dilutions of DNA and cDNA were spiked with 10^6^ copies of amplified CYP153 genes to check for real-time PCR inhibition. Highly linear standard curves (*r* ^2^ values >0.95, PCR efficiency >98%) over the dilution range and a detection limit of 10^1^ copies were obtained indicating no PCR inhibition. The CYP gene copy numbers were quantified relative to a standard curve of positive control and were normalized to the copy number of control plants [13], [26], [28].

### 6. Hydrocarbon analysis

Total petroleum hydrocarbons in soil samples were estimated by using FTIR as explained previously [20].

### 7. Statistical analysis

Plant growth, biomass production, residual hydrocarbon contents, gene abundance and expression data were analyzed by using Minitab software package. One-way analysis of variance (ANOVA) was used for the comparisons between treatments and Tukey's test was performed for ANOVA after testing homogeneity of variance.

## Results

### 1. Plant biomass

**Figure 1:**
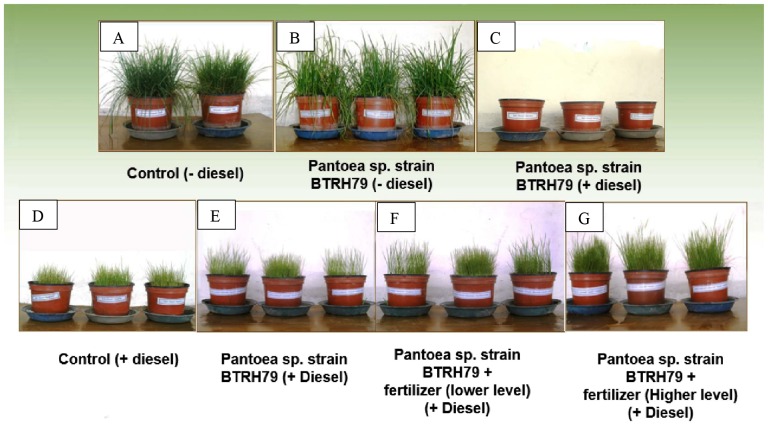
Experimental setup illustrating different treatments. Ryegrass vegetated in uncontaminated soil (A), ryegrass vegetated in uncontaminated soil and inoculated with *Pantoea* sp. strain BTRH79 (B), inoculation of *Pantoea* sp. strain BTRH79 in diesel contaminated soil (C), ryegrass vegetated in diesel contaminated soil (D), ryegrass vegetated in diesel contaminated soil and inoculated with *Pantoea* sp. strain BTRH79 (E), ryegrass vegetated in diesel contaminated soil treated with lower level of fertilize and inoculated with *Pantoea* sp. strain BTRH79 (F), and ryegrass vegetated in diesel contaminated soil treated with higher level of fertilizer and inoculated with *Pantoea* sp. strain BTRH79 (G).

Growth parameters (root fresh and dry biomass, shoot fresh and dry biomass, average shoot length, average shoot diameter and shoot network length) were estimated to assess the influence of nutrients and bacterial application on plant growth and biomass production (Tables 1 and 2, Fig. 1). In polluted soil, plants showed significantly less growth and biomass production than the plants vegetated in uncontaminated soil. However, bacterial inoculation and nutrients application enhanced plant growth and biomass production. Maximum plant growth and biomass production were obtained when inoculum was applied in the soil containing high concentration of nutrients. Bacterial inoculum in uncontaminated soil improved plant growth but not significantly.

**Table 1:** Effect of bacterial inoculation and nutrients on shoot length (SL), shoot and root fresh weight (FW) and dry weight (DW) of ryegrass vegetated in diesel contaminated soil after 100 days of sowing.

Treatment	SL (cm)	Shoot weight		Root weight	
		FW (g)	DW (g)	FW (g)	DW (g)
Control (− diesel)	27.6^a^ (1.6)	38.5^a^ (0.8)	8.5^a^ (0.4)	9.3^a^ (0.4)	3.94^a^ (0.03)
*Pantoea* sp. strain BTRH79 (− diesel)	29.1^a^ (1.3)	39.1^a^ (2.1)	8.8^a^ (0.2)	9.8^a^ (1.0)	4.15^a^ (0.04)
Control (+ Diesel)	7.9^d^ (0.6)	4.6^e^ (0.6)	1.7^d^ (0.1)	5.3^d^ (0.5)	1.45^d^ (0.03)
*Pantoea* sp. strain BTRH79 (+ Diesel)	15.1^c^ (1.7)	8.6^d^ (0.7)	2.2^d^ (0.1)	6.4^c^ (0.1)	2.90^c^ (0.08)
*Pantoea* sp. strain BTRH79 + fertilizer (lower level) (+ Diesel)	17.6^c^ (1.3)	10.9^c^ (0.4)	3.1^c^ (0.1)	7.6^b^ (0.4)	3.13^c^ (0.08)
*Pantoea* sp. strain BTRH79 + fertilizer (higher level) (+ Diesel)	21.7^b^ (1.6)	15.5^b^ (1.1)	4.2^b^ (0.5)	8.4^b^ (0.5)	3.61^b^ (0.04)

Each value is the mean of three replicates, means in the same column followed by the same letter are not significantly different at a 5% level of significance, and the standard error of three replicates is presented in parentheses.

**Table 2:** Effect of bacterial inoculation and nutrients on average shoot diameter and shoot network length of ryegrass vegetated in diesel contaminated soil after 100 days of sowing.

Treatments	Average shoot diameter (mm)	Shoot network length (cm)
Control (− diesel)	3.2^a^	112.8^a^
*Pantoea* sp. strain BTRH79 (− diesel)	3.3^a^	123.6^a^
Control (+ Diesel)	1.3^d^	32.4^d^
*Pantoea* sp. strain BTRH79 (+ Diesel)	1.8^cd^	65.6^c^
*Pantoea* sp. strain BTRH79 + fertilizer (lower level) (+ Diesel)	2.1^bc^	83.1^b^
*Pantoea* sp. strain BTRH79 + fertilizer (higher level) (+ Diesel)	2.4^b^	96.4^b^

Each value is the mean of three replicates, means in the same column followed by the same letter are not significantly different at a 5% level of significance, and the standard error of three replicates is presented in parentheses.

### 2. Abundance of inoculant strain and expression of alkane degrading gene

The survival and metabolic activity of the inoculated bacterium, *Pantoea* sp. strain BTRH79, in the rhizospheric soil were determined by both culture-dependent and independent approaches (Table 3). In uncontaminated soil with vegetation and in contaminated soil without vegetation, very poor survival and activity of the inoculated strain was observed. However, in contaminated soil, BTRH79 exhibited better colonization, and maximum colonization was observed when nutrients were applied in high concentration. Similarly, higher levels of the abundance and expression of CYP153 gene were found in the rhizospheric soil applied with higher concentration of nutrients.

**Table 3:** Effect of nutrients on CFU, abundance and expression of *Pantoea* sp. strain BTRH79 in the rhizosphere of ryegrass.

Treatment	CFU g^−1^ soil ×10^4^	Gene abundance (copies g^−1^ dry soil) ×10^4^	Gene expression (copies g^−1^ dry soil) ×10^4^
*Pantoea* sp. strain BTRH79 (+ diesel) (unvegetated)	0.62 (0.23)^d^	0.42^d^ (0.18)	0.16^d^ (0.11)
*Pantoea* sp. strain BTRH79 (− diesel) (vegetated)	0.73 (0.25)^d^	0.24^d^ (0.14)	0.07^d^ (0.02)
*Pantoea* sp. strain BTRH79 (+ diesel) (vegetated)	1801 (240)^c^	1230^c^ (371)	590^c^ (634)
*Pantoea* sp. strain BTRH79 (+ diesel) + fertilizers (lower level) (vegetated)	2780 (380)^b^	2040^b^ (259)	1230^b^ (213)
*Pantoea* sp. strain BTRH79 (+diesel) + fertilizers (higher level) (vegetated)	4015 (830)^a^	2750^a^ (455)	1860^a^ (462)

Each value is the mean of three replicates, means in the same column followed by the same letter are not significantly different at a 5% level of significance, the standard error of three replicates is presented in parentheses.

### 3. Hydrocarbon degradation

In planted soil, more hydrocarbon degradation was found than unplanted soil. Hydrocarbon degradation was further increased by bacterial and nutrients application. Maximum hydrocarbon degradation was found in the vegetated inoculated soil containing higher levels of nutrients (Table 4).

**Table 4:** Effect of inoculum and nutrients on hydrocarbon degradation.

Treatment	Hydrocarbon	
	Initial (g kg^−1^ soil)	Residual (g kg^−1^ soil)
Control (unvegetated soil)	15	12.54**^a^** (1.36)
Control (vegetated)	15	9.39**^c^** (0.99)
*Pantoea* sp. strain BTRH79 (unvegetated soil)	15	12.01**^b^** (1.81)
*Pantoea* sp. strain BTRH79 (vegetated)	15	5.25**^d^** (0.71)
*Pantoea* sp. strain BTRH79 + fertilizers (lower level) (vegetated)	15	3.83**^e^** (0.65)
*Pantoea* sp. strain BTRH79 + fertilizers (higher level) (vegetated)	15	2.04**^f^** (0.52)

Each value is the mean of three replicates, means in the same column followed by the same letter are not significantly different at a 5% level of significance; the standard error of three replicates is presented in parentheses.

## Discussion

**Figure 2:**
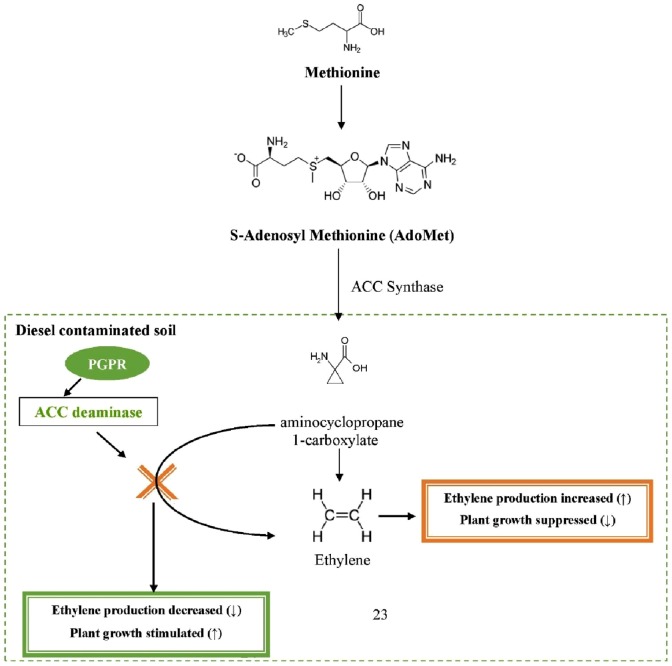
Schematic representation of how bacteria containing ACC deaminase activity lower the ethylene concentration and thereby prevent ethylene-caused inhibition of root elongation.

In this study, diesel contamination in soil reduced plant growth and development. It is well established that the presence of hydrocarbons adversely affects plant health and development due to their toxicity and hydrophobicity [4], [29]. However, when bacterial strain, *Pantoea* sp. BTRH79, was inoculated in diesel contaminated soil, it enhanced plant growth and development. Enhanced plant growth and development might be attributed to plant growth promoting activities (including ACC deaminase) of the inoculated bacterium [20], [21], [27]. In principle, bacterial ACC deaminase activity reduces the contaminant induced stress symptoms in developing plant and improves plant health and growth in the presence of contaminants as shown in Fig. 2 [30]–[32]. Similarly, hydrocarbon-degrading activity of the inoculated microorganisms reduces the phytotoxicity of pollutants due to their potential to produce hydrocarbon-degrading enzymes [33]. Plant growth was further enhanced by the application of nutrients. Earlier studies also reported that the application of nutrients improved plant growth and development during remediation of polluted soil [18], [34].

**Figure 3:**
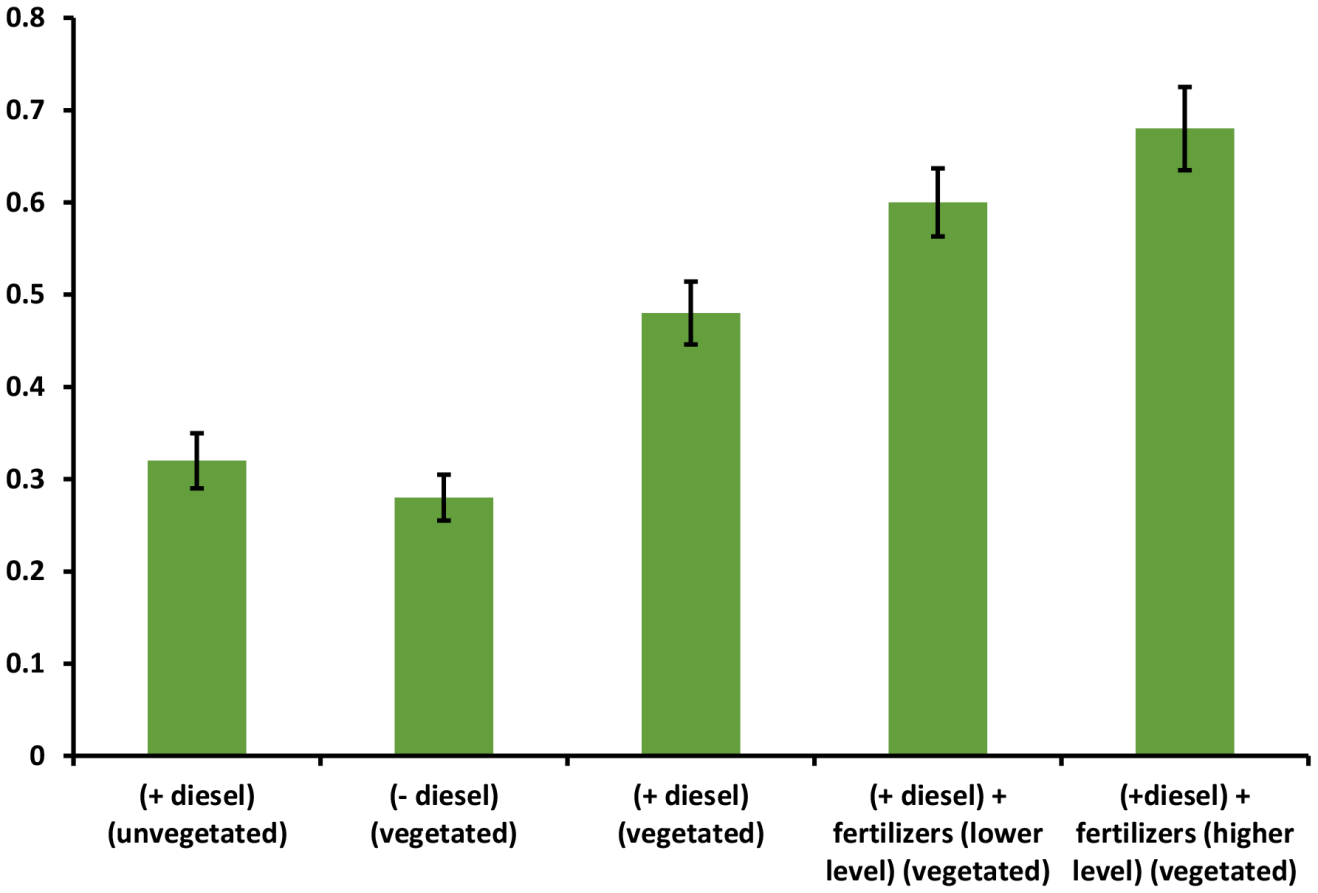
Ratio of gene expression and gene abundance in the vegetated and unvegetated soil.

The higher levels of bacterial colonization, the abundance and expression of CYP153 gene were observed in the rhizospheric soil of ryegrass vegetated in diesel-contaminated soil. A strong positive correlation (*r* = 0.991) between gene abundance and expression further indicated that the inoculated bacterium not only colonized but was also metabolically active in hydrocarbon degradation. In contaminated soil without vegetation, the survival of *Pantoea* sp. strain BTRH79 and expression of CYP153 were significantly lower than in the vegetated soil. This might be due to the fact that the inoculated rhizobacterium could not proliferate and was not metabolically active in the soil without vegetation. Nutrients application enhanced bacterial survival, the abundance and expression of CYP153 gene in the rhizosphere. Similarly, in a previous study, the addition of N, P and K in a petroleum-contaminated soil enhanced microbial population and hydrocarbon mineralization [35]. Between two levels of applied nutrients, significantly more gene abundance and expression were seen in the rhizosphere of ryegrass grown in the soil treated with higher level of nutrients. Higher amount of applied nutrients was required for bacterial proliferation and their catabolic activity [36]. The ratio between gene expression and gene abundance suggests that there were more metabolically active cells in the presence of high concentration of nutrients in the soil (Fig. 3).

Similarly, more hydrocarbon mineralization was found in the vegetated soil than the unvegetated soil. It is well known that plants enhance population and pollutant-degrading activity of microorganisms in soil [37], [38]. Hydrocarbon degradation was increased with bacterial inoculation and it might be attributed to hydrocarbon-degrading and plant growth-promoting activity of the inoculated strain, *Pantoea* sp. BTRH79. Many earlier studies have also reported that the application of pollutant-degrading and plant growth-promoting bacteria increased hydrocarbon degradation [6], [21]. In this study, hydrocarbon mineralization was further increased by the application of nutrients (N, P and K) in addition to inoculum. Between two applied nutrients levels, more hydrocarbon degradation was observed with higher nutrients level. This might be due to the fact that higher amount of nutrients enhanced more plant growth and microbial population in the rhizosphere that consequently lead to more hydrocarbon degradation.

Statistically speaking, a strong positive correlation (*r* = 0.934) was found between plant biomass and hydrocarbon degradation. This supports the idea of high degradation if we manage to increase the plant biomass. Furthermore, regression estimation illustrates that the increment of one unit of hydrocarbons statistically reduce the plant biomass up to 3.007 grams. Plant response (*Y*) in the presence of hydrocarbons can be written as follows:
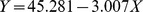



Hereby, 45.281 is the statistically calculated value of plant biomass when there was no diesel in the soil. Therefore, adjustment of the nutrients can optimize the remediation setup and improve the plant biomass that ultimately results into the degradation of petroleum hydrocarbons.

## Conclusions

This study demonstrates that the nutrients level in soil affect plant growth, hydrocarbon degradation and bacterial colonization and activity. Nutrients can enhance the survival of inoculated bacteria, the abundance and expression hydrocarbon-degrading gene in the rhizosphere. Therefore, the combined use of pollutant-degrading bacteria and nutrients is a more promising approach to enhance phytoremediation efficiency. Further studies should be conducted to determine the individual role of these nutrients as well as the optimum dose of nutrients with a specific plant to get maximum bacterial colonization and activity of pollutant degrading genes.
